# Sjögren Syndrome: New Insights in the Pathogenesis and Role of Nuclear Medicine

**DOI:** 10.3390/jcm11175227

**Published:** 2022-09-04

**Authors:** Anzola Luz Kelly, Rivera Jose Nelson, Ramírez Sara, Signore Alberto

**Affiliations:** 1 Nuclear Medicine Unit, Clinica Universitaria Colombia, Bogotá 111321, Colombia; 2 Nuclear Medicine Unit, Clinica Reina Sofia, Bogotá 110121, Colombia; 3 Fundacion Universitaria Sanitas, Bogotá 110111, Colombia; 4 Internal Medicine Department Clinica Reina Sofia, Bogotá 110121, Colombia; 5 Nuclear Medicine Unit, Department of Medical-Surgical Sciences and of Translational Medicine, Faculty of Medicine and Psychology, “Sapienza” University, 00185 Rome, Italy

**Keywords:** Sjögren syndrome, nuclear medicine, radiopharmaceuticals, PET, SPECT

## Abstract

In the last years, new insights into the molecular basis of rheumatic conditions have been described, which have generated particular interest in understanding the pathophysiology of these diseases, in which lies the explanation of the diversity of clinical presentation and the difficulty in diagnostic and therapeutic approaches. In this review, we focus on the new pathophysiological findings for Sjögren syndrome and on the derived new SPECT and PET radiopharmaceuticals to detect inflammation of immunological origin, focusing on their role in diagnosis, prognosis, and the evaluation of therapeutic efficacy.

## 1. Introduction

Sjögren syndrome (SS) belongs to the family of rheumatic autoimmune diseases characterized by systemic compromise with exocrine glands as target organs that are affected by chronic inflammation and immune-mediated destruction of the tissue, leading to severe dryness of the mouth and eyes. Extra-glandular symptoms are frequent and include fatigue, polyarthralgias, myositis, polyneuropathy, and gammaglobulinopathies, among others [1]. Since there is evidence of the presence of common pathophysiologic mechanisms shared by SS and thyroiditis, it is also common to find thyroid involvement in patients with SS as part of the peri-epithelial extra salivary manifestations of the disease [2]. Systemic complications regarding extra-glandular tissues are associated with vascular, respiratory, gastrointestinal, renal, and neurological systems, possibly affecting one-third of these patients [1].

SS may be present as a primary condition or accompanying other autoimmune diseases as secondary SS. In addition, SS has a predominant clinical presentation in females, with a female:male ratio of 9:1, higher than for all other autoimmune diseases [1]. The clinical phenotype of SS varies from benign conditions such as mild exocrinopathies to severe systemic manifestations such as B-cell non-Hodgkin’s lymphoma (NHL) [3,4]. These lymphomas arise predominantly from memory B cells in the marginal zone of lymphoid tissue, with mucosa-associated lymphoid tissue (MALT) lymphomas being the most frequent type [5] and the parotid and minor salivary glands being the most frequent sites of involvement [6]. Patients at high risk of developing NHL are male and have clinical manifestations of severe systemic disease, such as vasculitis, splenomegaly, lymphadenopathy, glomerulonephritis, haematologic manifestations, high expression of autoantibodies, cryoglobulins and hypergammaglobulinemia, high biopsy focus scores, and presence of ectopic germinal centers, among others [7].

## 2. Pathogenesis

The pathogenesis of SS is complex, some fundamental concepts are in construction and are still a matter of controversy; the lack of hard evidence has not been a barrier for different authors to approach the subject through theoretical models, which have been based on the results of observational studies in humans and in preclinical data.

**a.**  **Etiology**

Several factors have been implicated in the etiology of SS, as follows:

- Genetic components: The importance of type I interferon has been recognized in the pathogenesis of SS. In pSS, labial salivary gland and peripheral blood gene expression microarray studies, have demonstrated dysregulation of type I interferon-inducible genes [8]. The Genome-Wide Association studies (GWS) reported a strong association in SS within the HLA region at 6p21 (OR = 3.5) and with IRF5 (transcription factor mediating type I interferon responses in monocytes, dendritic cells, and B cells that induces the transcription of interferon-alfa genes and the production of pro-inflammatory cytokines upon viral infection), STAT4, IL12A and TNIP1 loci [9]; although genetic factors determine the baseline disease susceptibility and disease phenotype, these factors contribute modestly to the clinical condition [10].
- Epigenetic mechanisms: Different epigenetic mechanisms, such as DNA methylation, histone modifications, and non-coding RNAs, are important factors for modulating gene expression and generating an important link between the genome and phenotypic manifestations [11].
- Biological factors: several different types of infections can increase the risk of SS, triggering a proinflammatory microenvironment that promotes autoimmunity. As it was mentioned above, the GWS reported a strong association with the presence of IRF5 loci and the transcription of interferon-alfa genes, and the production of pro-inflammatory cytokines upon viral infection [9]. Both type I IFN and virus TLR ligands can stimulate the production of BAFF (B cell-activating factor of the tumor necrosis factor family) in cultured salivary glands epithelial cells, suggesting that viral infection could be responsible for the increase in BAFF production by ductal epithelial cells in pSS [12]. Different viruses have also been implicated in SS pathogenesis, such as Epstein–Barr virus (EBV), cytomegalovirus, hepatitis C, human T-lymphocyte virus type I, and hepatitis B [13]. Especially reactivation of latent EBV in genetically and hormonally susceptible individuals could play a role in the initiation and perpetuation of the chronic inflammatory autoimmune response in the glands [14]. Current evidence supports the fact that viral infections are factors that largely increase the risk of SS [10]. To this respect, it is important to clarify that epidemiologic studies only confirm association but do not prove causation.

Other biological factors that have been related to the pathogenesis of SS lack solid evidence, such as vaccination (information from case reports), vitamin D deficiency (few studies with selection bias and confounding factors), stress, and hormones (few studies).

- Organic chemical factors: smoking, alcohol, solvents (prevalence studies with poor evidence) [15].
- Inorganic chemical factors: silicone breast implants and silica. In recent years, clinicians have become aware of the existence of autoimmune/inflammatory syndrome induced by adjuvants (ASIA) associated with previous agents such as vaccines and silicone implants [16]; the authors described that such implants may lead to heterogeneous symptoms such as body aches, abnormal fatigue, depression, dry eyes, dry mouth, and chronic fatigue syndrome, among others. In a meta-analysis conducted to determine long-term health outcomes in women with silicone gel breast implants (Oxford level of evidence III), the authors found an association between silicone implants and the risk of SS, but they highlighted the presence of information bias because the data were from studies on patients with the self-reported disease [17]. Thus, although the evidence is weak to date, the existence of an approach based on a theoretical model of Shoenfelds et al., which shows that silicone can enhance antigen-specific immune response, should be recognized. It is necessary to conduct validation studies in larger cohorts of patients as well as randomized trials.

**b.**  **Immune response pathways**

SS pathophysiology includes concurrent dysregulation of an innate and adaptative immune pathway involving cell-mediated and humoral disease processes that are incompletely understood [18].

- Adaptative immunity: The involvement of this immunity in SS pathogenesis is evident through observations of autoreactive T and B cells, with pronounced B-cell hyperactivity, which appears to be the cornerstone of the disease process. Signs of this condition are well documented in the literature, as clinical findings in different tissues of the body and the observations in serological and histopathological markers (salivary glands, saliva, tears, serum, peripheral blood B cells, intrinsic B cell abnormalities, and the presence of germinal center-like structures, elevated levels of B cell-associated cytokines and chemokines, presence of anti-SSA/SSB autoantibodies, hypergammaglobulinemia, elevated levels of soluble CD27, amongst others) [19]. In spite of the recognized role of the B cells in the pathogenesis of SS, its exact contribution is partly understood, most data have been obtained from mouse models and some authors have shown the presence of a coordinated and integrated stimulation of B-cell receptor B, CD40 and Toll-like receptors (TLRs) with different cytokines during the process [20].
- Innate immunity: In most pSS, type I interferon (IFN) and type I IFN-induced genes and proteins are overexpressed, resulting in the so-called type I IFN signature of pSS; aberrancies have been described in the type I IFN system in salivary glands of pSS [21] as well as abnormalities in type I IFN-alfa in labial biopsies [22]; the effect of type I IFN is not only local on the salivary glands but is also systemic because of its up-regulation activity which could explain the main extraglandular manifestations of the disease [23].

**Figure 1:**
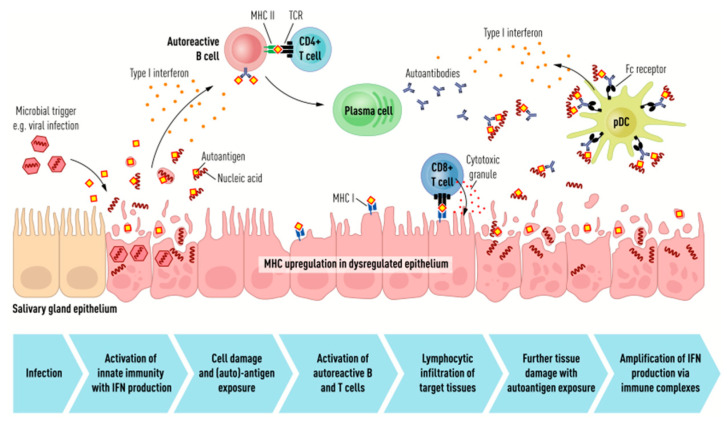
Schematic representation of the pathogenetic mechanisms at the basis of Sjogren syndrome. **IFN**: interferon. **pDCs:** plasmacytoid dendritic cells. **MHC**: major histocompatibility complex. **TCR:** T cell receptor. Reprinted with permission from Environmental factors in the pathogenesis of primary Sjögren’s syndrome, A. Björk, J. Mofors, M. Wahren-Herlenius., 2020, Journal of Internal Medicine, 287; 475–492.

Björk et al. have proposed a model to approach the possible pathogenic mechanisms associated with SS, which can be summarized as follows:

A trigger (e.g., viral infection) initiates disruption of the salivary gland epithelium, inducing the production of type I IFN, and auto-antigens released by the dying cells create an inflammatory microenvironment. Thereafter, antigen-presenting cells present viral and self-antigens, leading to the activation of autoreactive B and T cells with subsequent differentiation and activation of autoantibody-producing plasma cells. Tissue damage is induced by autoreactive T cells via the secretion of cytotoxic granules, disrupting the epithelium and amplifying exposure of the autoantigens. The autoantibodies and autoantigens form immune complexes that bind to receptors on plasmacytoid dendritic cells, enhancing the production of type I IFN, which promotes autoantibody production through the differentiation and activation of autoreactive B cells. Thus, a self-perpetuating cycle of autoimmunity is created (see Figure 1) [10].

**c.**  **Ectopic Lymphoid-Like Structures (ELSs)**

**Figure 2:**
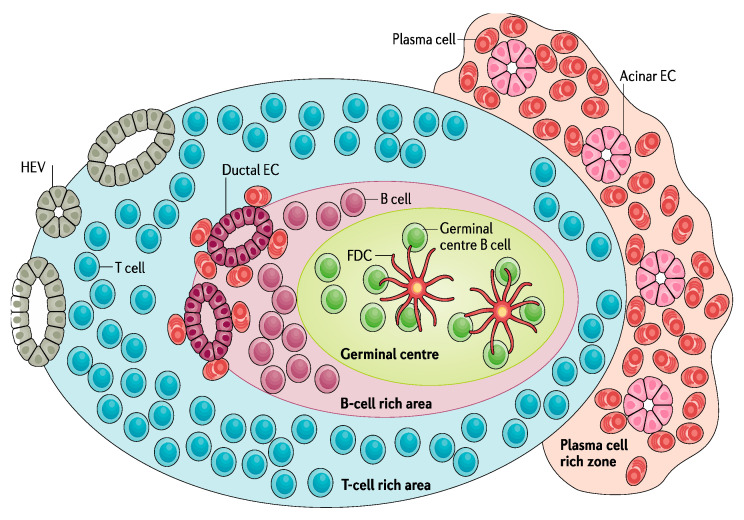
Schematic representation of an ectopic lymphoid-like structure (ELS), Segregation of T cells surrounding B cells. Development of high endothelial venules (HEVs) at the periphery of the ELS that enable lymphocytes expressing L-selectin to enter from the blood. Networks of follicular dendritic cells (FDCs) that support the germinal center response which expresses CD21 to facilitate the activation of B cells. Differentiation of hypermutated and class-switched plasma cells, which typically acquire a perifollicular localization with frequent infiltration of epithelial cells (ECs) from the ducts and acini. Reprinted by permission from Springer Nature Customer Service Centre GmbH: MDPI AG, Nature Reviews Rheumatology, Ectopic lymphoid neogenesis in rheumatic autoimmune diseases, Michele Bombardieri et al., COPYRIGHT, 2017.

Ectopic lymphoid-like structures (ELSs) play an important role in the pathogenesis of rheumatic autoimmune diseases. These structures belong to tertiary lymphoid organs, which are composed of clusters of mononuclear cells organized in target organs (non-lymphoid organs) at sites of chronic inflammation. It is known that ELSs can function as germinal centers, favoring B-cell selection and plasma cell differentiation and very often exhibiting an autoreactive phenotype to disease-specific autoantigens. A germinal center may be present within the structure, which in SS patients is associated with more severe systemic manifestations and a higher risk of B-cell lymphoma [24]. A schematic representation of an ELS is shown in Figure 2.

The estimated prevalence of ELSs in SS is 30–40%, frequently around central duct structures, leading to the belief that ELSs may play an important role in antigen recognition and induction of an immune response against ductal epithelial cells [25]. The mechanisms responsible for ELS formation are grouped according to four different moments of the development of the ELS as follows:

- Early activation of ELSs refers to the triggering process and depends on initiating factors.
- Regulation and maintenance of ELSs depend on propagating factors that regulate progression towards organized ELSs as well as their maintenance.
- Acquisition of the characteristics of a germinal center depends on functional factors.
- Survival maintenance of germinal centers by other factors; in this setting, it is important to highlight the presence of IL-27, which has a negative regulatory role and acts as an inhibitor of ELS development [26].

The most important initiating factors are the chemokines CXCL13, IL-7, IL-22, CCL21, CCL19, and RANK ligand, the signal of which regulates the migration and survival of lymphoid tissue inducer cells [27]. Previous reports have documented high expression of these chemokines in ELSs, in salivary glands of patients with SS, and in the synovium of RA patients [28]. Other proinflammatory cytokines have also been described as initiating factors, such as IL-23 and IL-17 [29]. The above concept supports the fact that cells of the adaptative immune system become capable of releasing key mediators that are crucial for the progression and maintenance of ELSs. Some chemokines and their cellular sources involved in ELS activation and organizational processes are summarized in Table 1 [24].

Factors considered mediators of ELS function are secreted by specialized T helper cell subsets that migrate into B-cell follicles, where the development of functional germinal centers requires a strong interaction between T and B cells [30]. Moreover, several factors, such as cytokines and membrane-bound ligands, are required to activate the function of these germinal centers; as a result of the strong interaction between T follicular helper cells (_TFHs_) and B cells, IL-21 is released. This cytokine is a potent cofactor for B-cell survival and proliferation and plasma cell differentiation [31] that has been implicated in the development of ELSs in rheumatic disease, particularly in SS and RA [32,33]. Indeed, in the salivary glands of SS patients, the interaction between CD4^+^ cells and activated epithelial cells promotes T_FH_ cell differentiation and IL-21 production [34]. Regarding facilitating factors, the role of IL-17 as a down-regulator of ELS development deserves mention, and it has attracted scientific interest as a potential factor for the treatment of rheumatic diseases via gene therapy [35].

The ectopic germinal centers of ELSs are associated with the local production of autoantibodies and have been associated with the maintenance of autoimmunity within a target organ. In SS patients with ELSs, the prevalence of circulating anti-Ro/SSA and anti-La/SSB antibodies is 20% higher than that in patients without ELSs [36]. ELSs promote affinity maturation and differentiation of plasma cells reactive against disease-specific autoantigens; these autoantigens are exposed as a result of the chronic inflammatory process and are presented to B cells and T cells by antigen-presenting cells.

Although the processes involved in the formation of ELSs in the synovium and in the salivary glands in AR and SS patients share common pathways, the antigen-driven autoimmune response appears to be disease-specific.

Under the pathological condition of rheumatic autoimmune disease, the autoreactive plasma cells generated in the ELSs are retained within the target tissue by the action of CXCL12, IL-6, and APRIL [37,38].

There is evidence that in SS patients, the presence of ELSs is associated with disease progression, high levels of circulating autoantibodies, and systemic manifestations, such as lymphadenopathy and peripheral neuropathy [36]. Indeed, there are reports of a strong relationship between the presence of ELSs in salivary gland biopsy and a higher risk of developing lymphoma [39].

Thus far, we have reviewed how the study of ELSs has allowed the investigators to build a theoretical model that undoubtedly will serve as a fundament for future investigations. Overall, a better understanding of the physiopathology and histopathological heterogeneity of target tissue in rheumatic conditions might lead to the identification of different molecules and markers to be used for diagnostic and therapeutic purposes, though clinical evidence from research with larger cohorts of patients and randomized studies is necessary.

The two most difficult situations in SS are as follows:

- the difficulty of early and accurate diagnosis, which is crucial to avoid major complications;
- a means of evaluating the efficacy of therapy.

To facilitate diagnostic and prognostic approaches for SS, several tools have been validated, such as the following:

- for diagnostic purposes: ACR-EULAR criteria [40],
- for calculating systemic disease activity: ESSDAI (Eular Sjögren Syndrome Disease Activity Index) [41],
- for patient-reported symptoms: ESSPRI (EULAR Sjogren Patient-Reported Symptoms) [41] and
- for evaluating response to different therapies: CRESS (Composite of Relevant Endpoints for Sjögren’s Syndrome) [42].

Despite these available tools, SS diagnosis remains underrecognized, with misdiagnosis occurring in some cases, impacting prognosis and the possibility of implementing the appropriate treatment.

The discouraging results in this field emphasize the heterogeneous characteristics of this systemic autoimmune disease and the urgent need to find better biomarkers that could elucidate the disease process for faster diagnosis (glandular function and the sicca symptoms are not always coupled) to provide better bases for further therapies.

## 3. Role of New Molecular Image Strategies in Sjogren’s Syndrome

The goal of SS is to be able to stratify pSS through the integration of clinical, laboratory, histopathology, and imaging data; thus, it could be possible to identify which patients have clinical manifestations related to inflammatory activity or sequelae; the possibility to identify which patients are on the risk of lymphoma development it is also important. Because new treatment strategies are emerging, there is also evident the need for new probes for a more reliable treatment response evaluation [43].

The potential for diagnosis and classification of pSS of the salivary glands ultrasound (SGUS) has been pointed out previously by different authors [44]; nowadays this technique is emerging as a complementary tool for biopsy purposes. In a recent study the authors reported that by adding SGUS as a minor item to ACR/EULAR criteria, the sensitivity could rise to 95.6% [45]. Other authors have reported that a combined positivity of SGUS and anti-SSA antibodies provides a high predictive value for the diagnosis of pSS [46] The main limitation of SGUS is the lack of a standardized scoring system, however, as an initiative to overcome this issue, the Outcome Measures in Rheumatology Clinical Trials (OMERACT) proposed a four-grade semiquantitative score with good intra and interobserver agreement results [47]. Last generation ultra-high resolution ultrasound (UHFUS) transducers, which produce frequencies up to 70 MHz with resolution up to 30 um, offer new possibilities to visualize labial salivary glands and to guide diagnostic biopsy procedures [48]. Recently, Baldini et al. demonstrated that the mean labial glandular surface area obtained by the high-resolution ultrasound-guided procedure was significantly higher than the area obtained by the traditional biopsy procedure. This procedure could facilitate the assessment of the focus score [49].

Promising innovations are ultrasound elastography (with algorithms to evaluate quantitatively the tissue stiffness of the salivary gland tissue [50], the application of artificial intelligence to automatically score for biopsy purposes [51], and the ultrasound-guided core needle biopsy of major salivary glands which could represent an alternative to surgical biopsy [52].

MRI does not belong to the common standard tools used for the diagnostic approach of pSS. Because of its high spatial resolution the major impact in such patients is for local staging of pSS associated with salivary glands lymphomas [53].

Salivary gland scintigraphy is a nuclear medicine technique that through the administration intravenously of ^99m^Tc-04 allows for to evaluate of the function of major salivary glands (perfusion, concentration, and elimination characteristics) [54]. Sialoscintigraphy is no longer part of the recent classification criteria for pSS [40]; the reported diagnostic approach shows a sensitivity of up to 90% with specificity of around 50%, making this tool not able to distinguish the functional compromise of SS and other salivary glands pathologies. It is possible that this technique could have some potential indication in the future as part of the tools available for the follow-up of patients [54].

Molecular nuclear medicine imaging has emerged with several biomarkers with potential clinical impact for its contribution to diagnosing, assessing the inflammatory status, and assessing disease progression. These images allow in vivo the characterization of cells and the phenomena involved in inflammatory diseases. For this purpose, by using radiolabeled molecules (administered in nanomolar amounts), that participate in the biochemical and pathophysiological process of chronic inflammation, it is possible to make a qualitative and quantitative assessment of the inflammatory burden in vivo. Recent advances in the development of target-specific imaging agents, allow us to perform a non-invasive evaluation of various molecular events such as angiogenesis, apoptosis, and cell trafficking in living organisms. Cellular and molecular changes occur a long time before structural changes, therefore, non-invasive visualization and quantification of molecular processes facilitate the early detection of the disease, establish a prognosis, and could potentially estimate the potential impact of biologic therapies.

In this review, we focus on radiopharmaceuticals that provide in vivo pathophysiological information about the disease not only in the salivary glands but also in other tissues that may be affected as part of the spectrum of the SS, such as the joints and thyroid gland.

**a.**  **Somatostatin Receptor Imaging**

**Figure 3:**
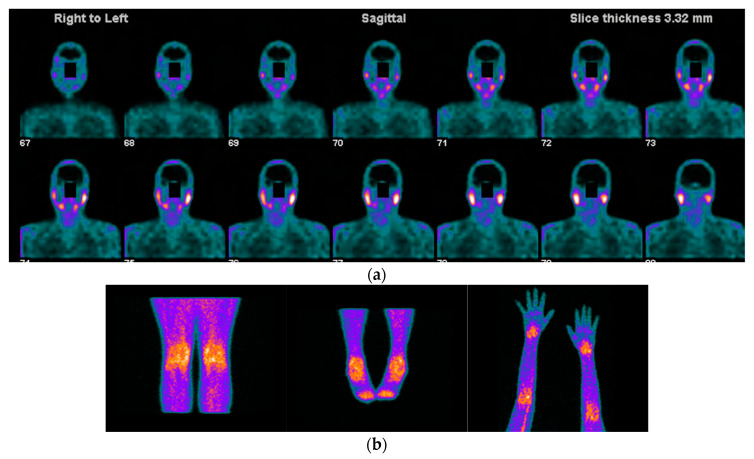
Images acquired after ^99m^Tc-HYNIC-TOC administration i.v in a patient with SS, sicca symptoms, and painful joints. In (**a**) high abnormal uptake in parotids and submandibular salivary glands. In (**b**) shows high abnormal uptake in knees, ankles, elbows, and carpal joints.

**Figure 4:**
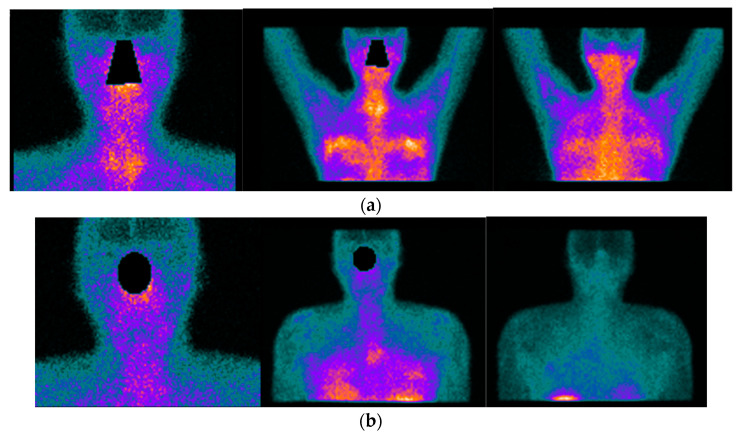
42-year-old female patient with the previous history of bilateral mammoplasty; four years later she complained of dryness (VAS 5/10), fatigue (VAS 10/10), pain (VAS:8/10) ESSPRI: 23, and thyroiditis. She was diagnosed with SS under ACR-EULAR criteria and she started treatment with DMARDS for two years without relieving symptoms. She asked her surgeon to remove the implants and six months after the removal of the implants there was an improvement in her clinical condition, dryness (VAS: 2/10), fatigue (VAS:6/10), pain (VAS: 5/10) post-surgery ESSPRI: 13. In (**a**) ^99m^Tc HYNIC-TOC images show high abnormal uptake of the radiotracer in the thyroid gland, submandibular glands, with a high abnormal bilateral mammary periprosthetic distribution of the radiotracer. SS was diagnosed under ACR-EULAR criteria. In (**b**) ^99m^Tc HYNIC-TOC images 6 months post removal of prosthesis show a significant decrease in uptake of the radiotracer in submandibular glands and thyroid gland. There was a concern that silicone could be associated with SS.

Radiolabelled peptides are highly specific and are used to reveal the presence of target molecules in inflammatory disease through molecular imaging. The peptides used in nuclear medicine images are easily synthesized, stabilized, and modified with good pharmacokinetic parameters; they also show high receptor binding affinity and are internalized into cells [55]. One of the most commonly used radiolabelled peptides for the inflammatory disease clinical approach is somatostatin, which has been used for inflammatory diseases for more than two decades, particularly in rheumatoid arthritis, SS, and autoimmune thyroid disease [56,57]. This is supported by the well-known presence of active and over-expressed somatostatin receptors in inflammatory and immunological cells from different tissues [58]. The diagnostic accuracy is also high because of the strong binding affinity of somatostatin to its five receptors. Different radiopharmaceuticals for somatostatin receptor scintigraphy are available, such as ^68^Ga-DOTA-TATE, ^68^Ga-DOTA-TOC, ^68^Ga-DOTA-NOC, and ^99m^Tc-EDDA/HYNIC-TOC [59], offering the possibility of detecting active inflammation in tissues affected by SS. Some of the evidence that supports the use of these radiopharmaceuticals in SS has been provided by Anzola et al., who reported the normal biodistribution of ^68^-Ga-DOTA-NOC and reference values for salivary glands, thyroid glands, and major joints, constituting the starting point for PET studies for further analysis in patients affected by rheumatic inflammatory disorders [60]. Using a cohort of 62 patients with confirmed SS by AECG criteria, the same group [61] reported the ability of ^99m^Tc-HYNIC-TOC scintigraphy to identify active inflammatory processes not only in salivary glands but also in many joints. This work highlighted the capability of the molecule to evaluate inflammatory compromise in sites different from the salivary glands, to evaluate the inflammation status of the salivary glands, and to hypothesize the usefulness of the probe to assess response to treatment. In a pilot study in 18 patients with rheumatoid arthritis and secondary SS who received infliximab as treatment, the authors [62] showed uptake of ^99m^Tc HYNIC-TOC in all affected joints and in the salivary glands in 12 of 18 patients. They also demonstrated a significant reduction in uptake by joints but not salivary glands after treatment with infliximab, enhancing the potential of the molecule to assess disease activity in rheumatoid arthritis and to detect secondary SS (Figure 3 and Figure 4). It is important to highlight that because the synovia of patients with SS and rheumatoid arthritis highly express somatostatin receptors [63], radiolabelled somatostatin can identify sites of active inflammation in joints accompanying salivary gland compromise in primary or secondary SS.

One systematic review [56] reported promising results of radiolabelled somatostatin analogs as diagnostic tools for localizing and identifying sites of active inflammation in joints as well as extra-articular involvement, such as the salivary glands, in patients with chronic inflammatory diseases (Figure 3a,b and Figure 4a,b).

**b.**  **B-lymphocyte Imaging in SS**

Currently, the importance of the role of B lymphocytes in the pathogenesis of rheumatic inflammatory diseases through the production of auto-antibodies, T-cell activation, and pro-inflammatory cytokines is well recognized [64]. We previously reviewed how these cells are found in pathological infiltrates of affected tissues and are implicated in disease progression, with B-cell hyperactivity being fundamental to the disease. The maturation of B cells from stem cells suggests several steps, with changes in cell surface markers. Indeed, these surface markers have gained attention for use in B-cell-depleting therapies through the use of different monoclonal antibodies against them, acting directly (CD19, CD20, CD22) or indirectly via blockade of cytokine pathways (TNF-alpha, interleukin-6, B lymphocyte stimulator (BLyS) and proliferation-inducing ligand APRIL) [65,66]. Although some molecules are commonly used, scarce evidence is available, and the results of trials are inconclusive. No biologic drug has yet been approved for the treatment of pSS, though three biologics, i.e., rituximab, belimumab, and abatacept, have shown effectiveness in open studies for extra-glandular symptoms but not for dryness [67]. Anti-tumor necrosis factor-alpha (TNF-alpha) drug trials in pSS have failed to show promising results; although a potential protective role for TNF-alfa against lymphoproliferation has been hypothesized, it has been demonstrated that depletion of TNF-alpha may increase BAFF (B-cell activating factor) levels in humans, discouraging its use for pSS in clinical practice [68]. Rituximab is a chimeric monoclonal antibody directed against CD20, which is expressed on membrane B cells. Expert opinion [69,70,71] supports its use as a therapeutic option in pSS with systemic compromise and as second-line therapy for hematological manifestations. In addition, a radiolabelled anti-CD20 mAb probe for in vivo imaging of CD20-positive lymphocyte infiltration in inflammatory diseases has been described, offering the possibility of a better approach to disease staging [72].

In vivo imaging of lymphocyte B through the use of anti-CD20 radiotracer has potential applicability for SS patients. Malviya et al. [73] used radiolabelled anti-CD20 (rituximab) and showed B Lymphocyte infiltration in affected tissues of patients with different chronic inflammatory autoimmune diseases and who were candidates for rituximab treatment. The authors reported the in vivo localization pattern of the B-lymphocytes mediating the inflammatory process, with variable uptake by salivary glands and lacrimal glands in two SS patients. Despite the small sample size, this work provides the basis for further studies assessing whether an antibody accumulates in inflamed tissue before using the same antibody for therapeutic purposes, thereby improving the selection of patients who might benefit from the therapy, which is called immune scintigraphy for therapeutic decision-making.

**c.**  **T-lymphocyte Imaging in SS**

Because activated T lymphocytes are involved in chronic inflammatory diseases and IL2 is mainly secreted by these cells to play a regulatory role during inflammation, some authors have developed a means of detecting these cells in vivo through the synthesis of different radiopharmaceuticals for SPECT and PET systems [74]. Reports to date have demonstrated its usefulness in different clinical inflammatory scenarios, such as in insulitis in type 1 diabetes [75], Crohn’s disease [76], and Hashimoto thyroiditis, among others, for diagnostic and prognostic purposes and for therapy follow-up.

**Figure 5:**
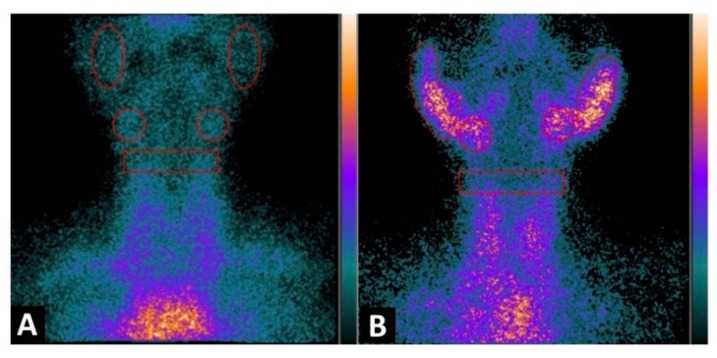
Planar image of the neck was obtained 1h after ^99m^Tc-IL2 injection in a control subject (**A**) and in a patient with Sjögren syndrome at the time of diagnosis (**B**). In (**A**) the scan shows no ^99m^Tc-IL2 uptake by the salivary glands. In (**B**) an evident accumulation of ^99m^Tc-IL2 can be observed in both parotids and submandibular glands, indicating the presence of activated lymphocytes. The calculated parotid to background (P/B) ratios are 1.35 and 1.30 in right and left glands, respectively, and the submandibular gland to background (S/B) ratios are 1.57 and 1.64 in right and left glands, respectively. Reprinted with permission from Imaging Activated-T-Lymphocytes in the Salivary Glands of Patients with Sjögren’s Syndrome by 99mTc-Interleukin-2: Diagnostic and Therapeutic Implications Campagna, G.; Anzola, L. K.; Varani, M.; Lauri, C.; Gentiloni Silveri, G.; Chiurchioni, L.; et al. *J. Clin. Med.* **2022**, *11* (15), 4368. [77].

In a recently published study [77], in a cohort of 48pSS, the authors reported the utility of ^99m^Tc-IL2 for evaluating in vivo the extent and severity of lympho-mononuclear cell infiltration in the salivary glands. When they compared the results with a control group they found a statistically significant difference (*p* < 0.0001) in the radiotracer uptake in salivary glands between the two groups; they also observed that the uptake in patients with a longer history of the disease was lower compared with the recently diagnosed patients. They highlighted the capacity of the molecule to detect active inflammation mediated by IL2 and the possibility to treat those patients with immune-modulatory drugs and using the probe for evaluating the efficacy of the treatment (Figure 5).

New radiotracers, such as ^18^F-fluorbenzoyl interleukin-2, have also been developed for PET systems, allowing detection of activated T lymphocytes for the same purposes. In a preclinical study, Di Gialleonardo et al. [78] identified activated T lymphocytes in inflamed tissues, highlighting the potential of the probe for detecting activated T lymphocytes in autoimmune diseases such as SS. Because T lymphocytes are also mainly implicated in the pathogenesis of SS, molecular images for detecting T lymphocyte activity would play an important role in diagnostic approaches [44].

**d.**  **Other PET Radiopharmaceuticals Used in SS**

Recently, in an age- and sex-matched study with SS patients and healthy volunteers using MRI, ^11^C-MET-PET, and ^18^F-FDG-PET, Jimenez-Royo et al. [79] reported how molecular imaging findings correlate with disease characteristics, providing information about the inflammatory and functional status of the salivary glands. ^11^C-MET PET (a protein synthesis marker incorporated into cellular proteins) information [80] was used to evaluate residual salivary gland function, and ^18^F-FDG PET information was used to evaluate the inflammatory condition through glucose utilization by the inflamed cell [81]. Their main findings were significantly lower ^11^C-MET uptake in the parotid and submandibular glands in SS patients than in volunteers and higher uptake of ^18^F-FDG in the salivary glands of SS patients, indicating the presence of inflammation. Furthermore, a negative correlation between ^11^C-MET and ^18^F-FDG uptake (loss of function with highly inflamed tissues) was reported. Regarding histological analysis, the most relevant finding was related to a moderate positive correlation between ^18^F-FDG uptake in the parotid gland and CD20+ B-cell infiltration in the minor salivary gland of patients, suggesting that ^18^F-FDG uptake shows B-cell tissue infiltration By using ^18^F-FDG PET in SS patients, Cohen et al. [82] found evidence of the added value of the tool, demonstrating systemic compromise in SS. These authors observed that although the most frequent site of uptake was the salivary glands, the lymph nodes and lungs were equally affected. They also described a PETC/CT inflammation score that correlated with ESSDAI and gammaglobulin levels, suggesting that ^18^F-FDG PET may help in assessing disease activity and represent an inflammation biomarker for SS.

Although interesting characteristics of ^18^F-FDG PET have been described as inflammation markers in SS, it is important to take into consideration that new probes would make possible more specific detection of inflammation by targeting specific molecules and cells expressed in compromised tissues, providing good characteristics not only for diagnostic purposes but also for therapy decision-making and follow-up [83].

Because primary SS may be associated with lymphoma with a risk of 5–7% [84] and this diagnosis might be difficult because of the common clinical findings with SS, the usefulness of ^18^F-FDG in the case of lymphoma for initial staging, biopsy guidance, evaluation of response to therapy and detection of relapse is well recognized [85]. Keraen et al. [86] compared ^18^F-FDG patterns in patients with active primary SS with lymphoma and those without lymphoma. They observed that salivary gland enlargement, particularly the parotid glands, was more frequent in lymphoma patients (*p* = 0.003) and that the presence of focal compromise in the lungs was highly suggestive of lymphoma (*p* = 0.01); they observed the highest SUVmax (maximum standardized uptake value) on whole scans in patients with lymphoma (*p* = 0.02); in ROC curve analysis, they reported a parotid gland cut-off > 4.7 on SUV max has a sensitivity and specificity of 73.3% and 86.7%, respectively, for the presence of lymphoma.

Other radiopharmaceuticals with the potential to detect inflammation in SS patients include ^68^Ga-pentixafor, a radioligand of the chemokine receptor CXCR4 that plays an important role in the trafficking of progenitor and inflammatory cells [87]. Cytawa et al. [88] demonstrated in a case report bilateral intense radiotracer uptake in the parotid and submandibular salivary glands, consistent with inflammatory cell infiltration in a patient with SS.

## 4. Conclusions

The more we investigate the pathogenesis of diseases, the more we understand how to prevent and treat them. However, we also define new important molecular targets for diagnostic purposes by tailoring new radiopharmaceuticals. This strategy certainly applies to rheumatic diseases and to SS in particular, and understanding the role of several molecules, receptors, and immune cells in SS has allowed the application of specific radiopharmaceuticals for targeting these molecules and cells in vivo. Furthermore, the availability of SPECT and PET studies has offered the possibility to show in vivo the presence, homing, and trafficking of different immune cells involved in the pathogenesis of SS, providing further understanding of the pathogenetic role of these cells. Current evidence supports conducting robust studies to determine the appropriate place for molecular imaging in the diagnostic flowchart of SS and for therapy decision-making and follow-up of therapy efficacy.

## Figures and Tables

**Table 1:** Cellular sources of the chemokines regulating ectopic lymphoid organogenesis.

Pathway	ELS-Positive Target Tissues in Rheumatic Autoimmune Diseases
CXCL13	CD4^+^ T Cells, CD14^+^ monocytes, CD68^+^ macrophages or DC, Endothelial cells, epithelial cells, fibroblast-like synoviocytes, and Follicular DCs.
CCL19	Myofibroblast-like stroma
CCL21	Myofibroblast-like stroma, Lymphatic endothelial cells, DCs.
RANKL	B cells, Fibroblast-like synoviocytes
IL-7	Fibroblast-like synoviocytes, Siblining synovial macrophages
IL-22	CD4^+^ T cells, NKp44 NK cells.

Abbreviations: CCL, CC: chemokine ligand; DCs: dendritic cells, NK: natural killer.

## Data Availability

Not applicable.
